# An imageology-based feasibility study of plating posterolateral tibial plateau fractures via an anterolateral trans-fibular-head approach

**DOI:** 10.1186/s12891-024-07311-6

**Published:** 2024-03-05

**Authors:** Xiaoji Zhou, Jiangshan Zhou, Huajun Qian, Chunxiao Qian, Bin Xu, Lv Pan, Xudong Chu

**Affiliations:** Department of Orthopedics, Affiliated Huishan Hospital of Xinglin College, Nantong University, Wuxi Huishan District People’s Hospital, Wuxi, Jiangsu Province China

**Keywords:** Imaging, Knee joint, Outcomes, Posterolateral fracture, Trans-fibular-head approach, Tibial plateau fractures involving posterolateral quadrant

## Abstract

**Background:**

There are many difficulties in the reduction and fixation of the tibial plateau fractures involving posterolateral quadrant using general plates via traditional approaches. By imaging the area above the fibulae capitulum, this study was performed to investigate the feasibility of the trans-fibular-head approach and to design an ideal anatomical plate.

**Methods:**

MRI and CT scans of the knee joint were collected from 205 healthy volunteers (103 males, 102 females). Gender and height were used to divide the volunteers into groups separately: (1) A1 group and A2 group according to gender, (2) B1 group and B2 group according to height. Based on the images, several parameters were defined and measured to describe the space above the head of the fibula. In addition, differences in these parameters between genders and height were compared.

**Results:**

The narrowest distance in the bony region was (10.96 ± 1.39) mm, (5.41 ± 0.97 mm) in the bone-ligament region. The narrowest distance of bony region in the A1 group was more than that in the A2 group (11.21 ± 1.62 mm, 10.85 ± 1.47 mm, *p* = 0.029). The narrowest distance of the bony region was (10.21 ± 1.42) mm and (11.65 ± 1.39) mm in the B1 group and B2 group, respectively (*p* = 0.002). The narrowest distance of the bone-ligament region was (5.39 ± 0.78) mm and (5.22 ± 1.21) mm in the A1 group and A2 group, respectively. No statistically significant differences were observed between the A1 group and the A2 group in terms of the narrowest distance of the bone-ligament region. In the B1 group, the narrowest distance of the bone-ligament region (5.18 ± 0.71 mm) was not significantly less than that (5.31 ± 0.91 mm) in the B2 group.

**Conclusion:**

The space above the fibular capitellum was ample enough to place the plate for treating tibial plateau fractures involving posterolateral quadrant. The divisions of the lateral tibial plateau by 3-dimensional CT and the parameters of each region were crucial for providing guidance for designing the anatomical plate for the trans-fibular-head approach.

## Background

**Fig. 1:**
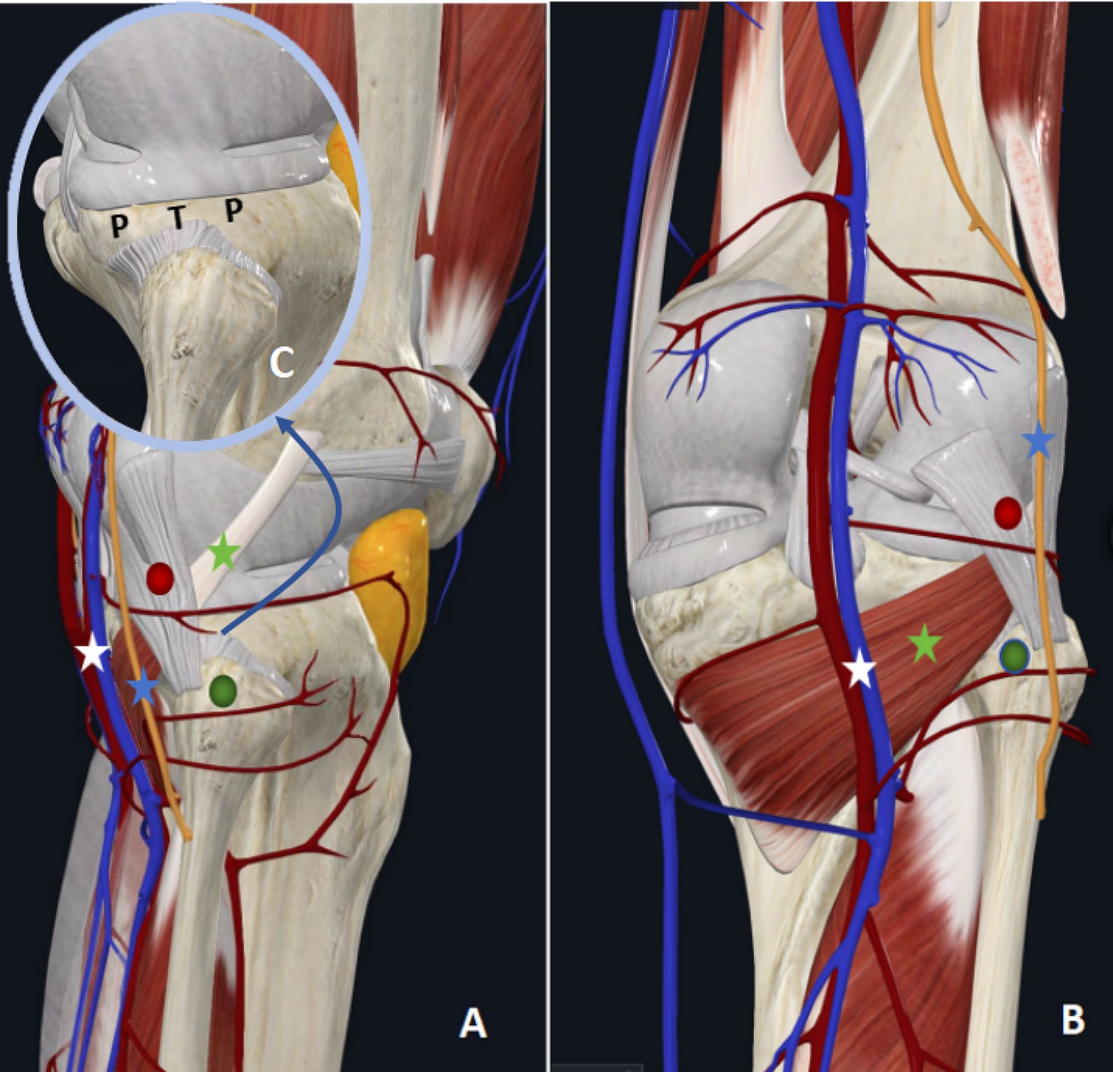
A three-dimensional illustration of the anatomical structures around the posterolateral tibial plateau. **A**, Lateral views showing the anatomical relationship. **B**, Posterior views showing the anatomical relationship. **C**, The space above the caput fibulae could be fully exposed after the fibular collateral ligament and hamstring tendon were retracted posteriorly. Green pin, caput fibulae; Red pin, fibular collateral ligament; Blue pentagram, common peroneal nerve; Green pentagram, musculus popliteus; White pentagram, vena poplitea and arteria poplitea; PTP, posterolateral tibial plateau

Tibial plateau fractures involving posterolateral quadrant, whether combined with another quadrant, are not as uncommon as previously thought [1–3]. Fractures of the posterolateral tibial plateau are primarily associated with high-energy trauma and require more attention when creating a preoperative plan. The most important surgical objectives for such injuries are optimal reduction and firm fixation. The traditional anterolateral approach is simple with which traumatic orthopedic surgeons are all familiar. However, it is challenging to expose and treat the fracture using conventional procedures through the traditional anterolateral approach since the posterolateral fragments are typically blocked by the fibulae capitulum and posterolateral corner structure. Figure 1 shows the anatomical structures around the posterolateral tibial plateau. In addition, proximal fibular osteotomy, which carries the risk of nonunion or malunion at the osteotomy site, was introduced for increased intraoperative exposure in the literature [1, 4]. Alternatively, a posterior approach is commonly described in the literature for treating posterolateral tibial plateau fractures. This approach necessitates the anatomical separation of bundles of blood vessels and nerves (the popliteal artery/vein, the tibial nerve, the common peroneal nerve, etc.) [5–7]. Nevertheless, this approach involves a high risk of iatrogenic damage to the local vasculature and nervus peroneus communis [8, 9].

In previous studies, our team proposed a novel anterolateral approach (the trans-fibular-head approach) specifically for fractures of the posterolateral tibial plateau, which has been proven to be anatomically feasible in human cadavers and effective in clinical practice [10, 11]. In recent years, we have also applied this trans-fibular-head approach for treating cases of posterolateral condylar fractures of the tibial plateau by using available market plates. However, we sometimes found that the space above the fibular-head was insufficient for a plate. Several studies have also proposed that there are no plates specific for the posterolateral fracture of the tibial plateau and that the available market anatomical plates are unable to be positioned posteriorly enough to support the posterior-lateral fragment [9, 12]. As Sassoon reported [13], 42% of the whole anteroposterior depth of the lateral tibial plateau could not be supported by using different plates from 5 famous manufacturers for treating posterolateral fractures of tibial plateau. Therefore, managing posterolateral tibial plateau fractures through a simple surgical approach with an applicable plate is a difficult challenge for traumatic orthopedic surgeons. To design an optimal periarticular plate for the trans-fibular-head approach and further support this surgical technique, in this paper, we investigated the detailed morphology of the supra-fibular-head space using magnetic resonance imaging and computed tomography in East Asians.

## Materials and methods

### General information

This study was approved by the Ethical Committee of the People’s Hospital of Huishan District (Ethics No.0001256), and written informed consent was obtained from all 205 healthy volunteers (mean age 47.3 ± 10.7 years, mean height 169.1 ± 9.7 cm), aged between 18 and 65 years. Gender and height were used to divide the volunteers into groups separately: (1) A1 group (103 male, 180 knees, mean age 45.5 ± 10.3 years, mean height 173.7 ± 10.2 cm); A2 group (102 female, 172 knees, mean age 49.1 ± 11.2 years, mean height 163.3 ± 7.6 cm) according to gender; (2) B1 group (156–170 cm, 165 knees, mean age 51.1 ± 10.9 years, 63 male, 49 female); B2 group (171 ~ 185 cm, 187 knees, mean age 44.9 ± 11.6 years, 40 male, 53 female) according to height. All methods were carried out based on relevant guidelines and regulations. MRI and CT imaging data from all volunteers were collected between July 5, 2020 and December 31, 2020. The following inclusion criteria were used: (1) no history of severe knee degeneration, knee injury, or knee operation, (2) imaging examinations and physical examination revealed no developmental abnormalities.

### Radiographical measurements

**Fig. 2:**
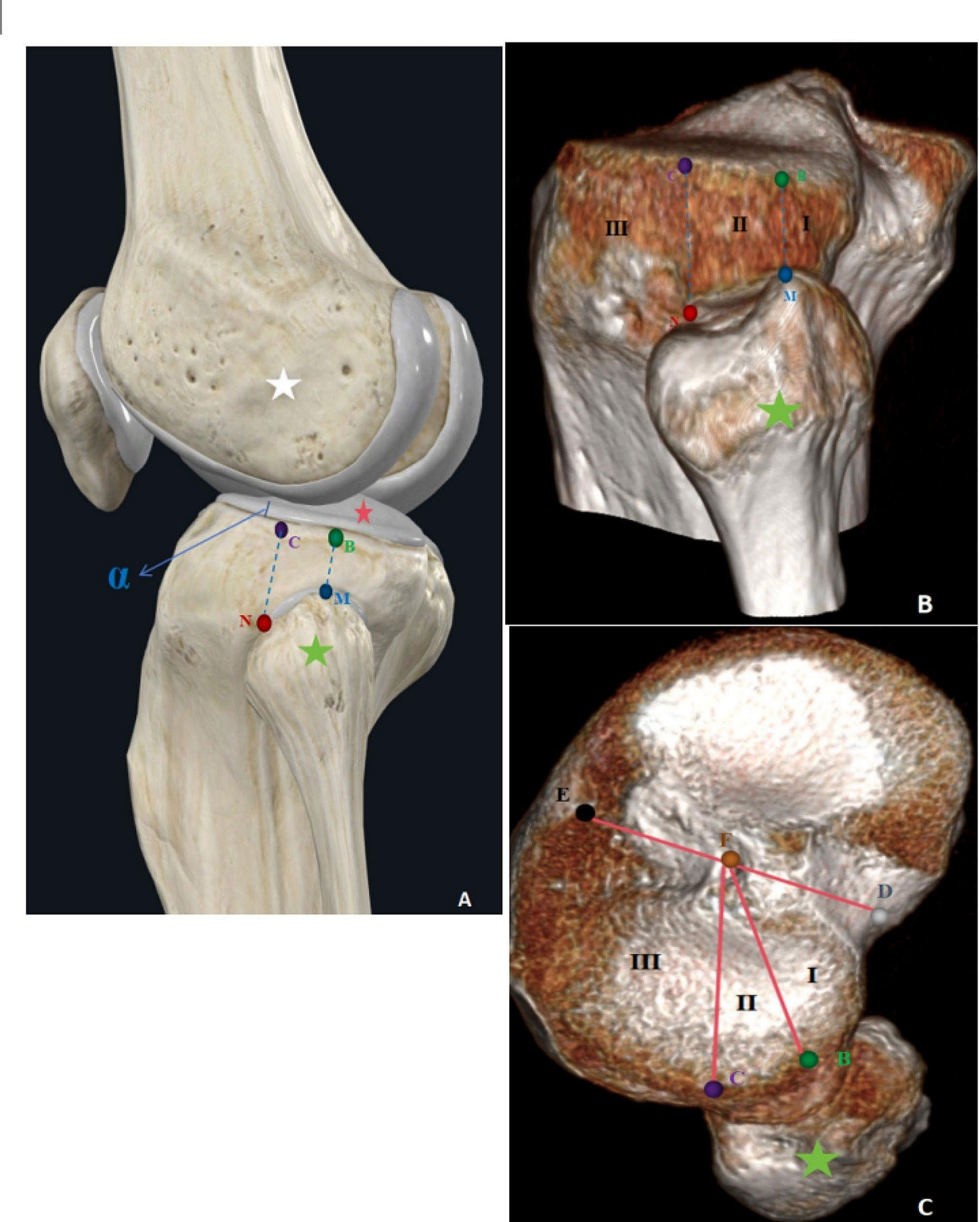
Schematic drawing of the bone region parameters of the supra-fibular-head space **(A)**. The actual measurement of the technique to measure the bone region parameters on the 3D CT image **(B, C)**. The blue pin indicates the superior border of the articular surface of the fibular head (M); the green pin indicates the vertical intersection of the blue pin to the lateral margin of the articular surface of the tibial plateau **(B)**; the red pin indicates the anterior border of the articular surface of the fibular head (N); and the purple pin indicates the vertical intersection of the red pin to the lateral margin of the articular surface of the tibial plateau **(C)**. I indicates region I; II, region II; and III, region III. The black pin indicates the centre of the leading edge of the tibial plateau **(E)**; the grey pin indicates the centre of the trailing edge of the tibial plateau **(D)**; the brown pin indicates the centre of the tibial intercondylar eminence **(F)**. α indicates the thickness of articular cartilage. Green pentagram, caput fibulae; White pentagram, lateral condyle femur; Red pentagram, articular cartilage

**Fig. 3:**
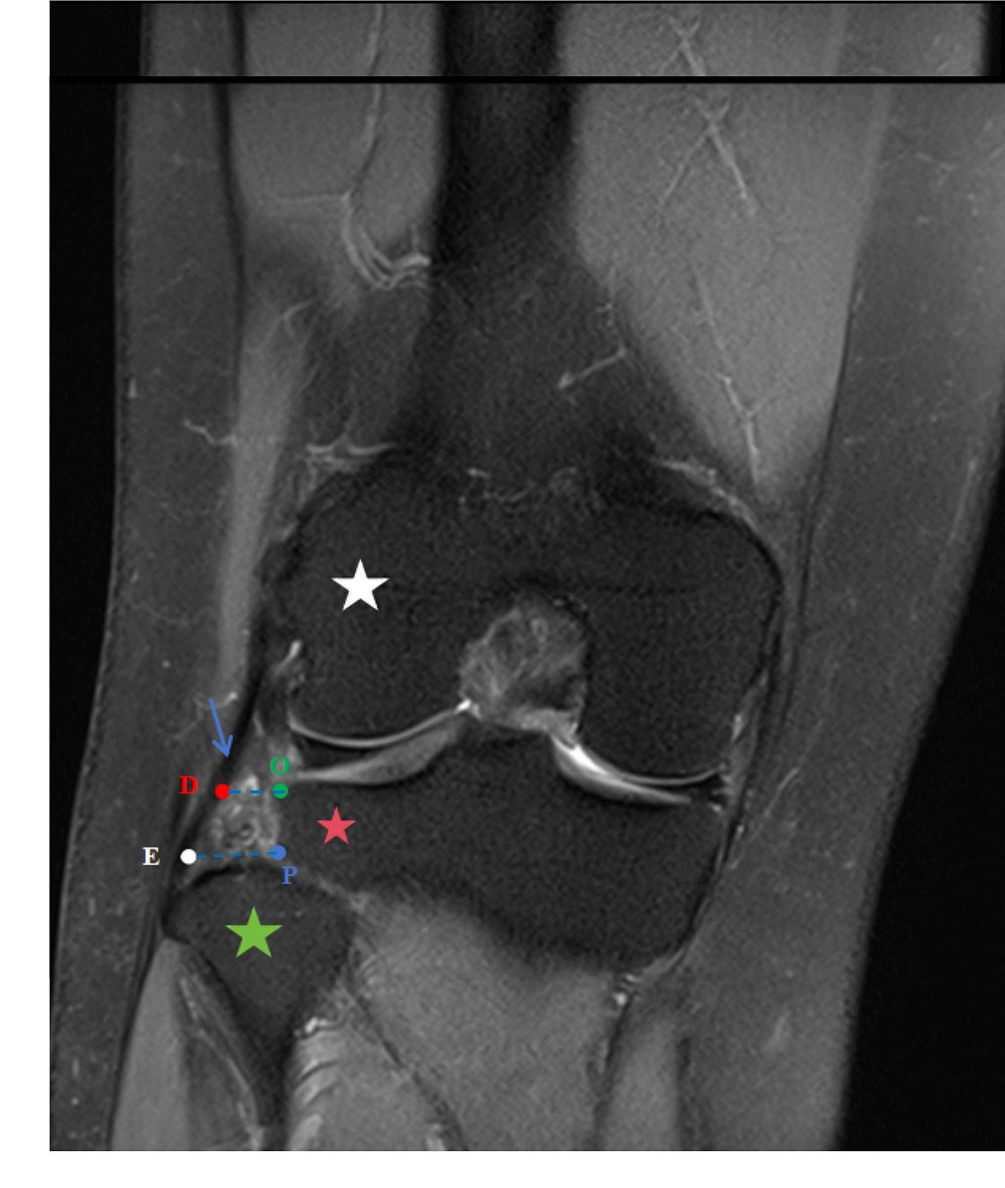
A MR coronal view of the space above the fibular head. The blue pin indicates the superior border of the articular surface of the fibular head (P); the white pin indicates the medial margin of the fibular insertions of the fibular collateral ligament (E); the green pin indicates the lateral margin of the lateral tibial plateau (O); and the red pin indicates the medial margin of the fibular collateral ligament (D). Green pentagram, caput fibulae; Red pentagram, the lateral tibial plateau; White pentagram, lateral condyle femur; Blue arrow, the fibular collateral ligament

We measured the parameters of the supra-fibular-head space on CT and MRI images. Knee joint CT images (CT 64-channel scanner; General Electric Company, America) were obtained intertemporally with the subject in a comfortable supine position and with the knees straightened. MR images of the knee joint were obtained using a 3-Tesla Siemens coil (Verio 3.0T, Siemens Corporation, Germany) via sagittal and coronal scanning. As shown in Figs. 2 and 3, the analyzed data from both imaging techniques included the parameters of the bony region, bone-cartilage region, and bone-ligament region.

#### Bony region parameters

The measurements included the following six indicators, which were obtained from the three-dimensional CT reconstruction images.

1. MB: The vertical distance from the superior border of the articular surface of the fibular head (M) to the lateral margin of the articular surface of the lateral tibial plateau (B).
2. NC: The vertical distance from the anterior border of the articular surface of the fibular head (N) to the lateral margin of the articular surface of the lateral tibial plateau (C).
3. BC: The vertical distance between line MB and line NC.
4. Region I: The area between the line MB and the tibial insertions of the posterior cruciate ligament, that is, the region of the posterolateral condyle of the tibial plateau.
5. Region II: The area of the supra-fibular-head between line MR and line NC, which is a trapezoidal frame.
6. Region III: The anterolateral area of the line NC, including the lateral surface of the middle and upper tibia.

#### Bone-cartilage region parameters

Since cartilage is not visible on CT scans, combined measures of the bone region parameters and cartilage thickness can be used to determine the parameters of the bone-cartilage region.

#### Bony-ligament region parameters

We selected the MR coronal tomographic image with the lateral collateral ligament for precise measurements.

1. PE: The distance between the superior border of the articular surface of the fibular head (P) and the medial margin of the fibula insertions of the fibular collateral ligament (E).
2. OD: The distance between the lateral margin of the lateral tibial plateau (O) and the medial margin of the fibular collateral ligament (D).

All imaging data were analyzed and studied by using SkyViewPACS V3.3.1.5 (Yangtze River Ruiheng Software, Ltd., Nanjing, China). Data on the trans-fibular-head region were measured by two radiologists with over 8 years of expertise and two orthopedic surgeons with over 10 years of experience.

### Data analysis

All the parameters were entered into Excel 2019 (Microsoft, Seattle, WA). The data analysis was performed using SPSS 26.0 software (SPSS Inc, Chicago, IL). The baseline continuous variables are presented as the mean ± standard deviation and were compared by using independent two-sample t-tests. *P* < 0.05 was considered to indicate statistical significance.

## Results

The line MB ranged from 7.7 to 12.8 mm, with a mean of (10.96 ± 1.39) mm. The line NC ranged from 13.8 to 18.1 mm, with a mean of (16.32 ± 1.49) mm. The line MB was significantly less than line NC (t = 8.968, *P* = 0.001). Line MB was the narrowest width in the bony region. The line BC ranged from 8.1 to 13.9 mm, with a mean of (9.90 ± 1.01) mm. The average line OD in the bone-ligament region was (5.41 ± 0.97) mm, ranging from 3.2 to 6.3 mm, which was narrower than that of line PE (9.11 ± 1.16 mm, range: 6.0-11.6 mm) (t = 9.125, *P* = 0.001). The narrowest width in the bone-ligament region was the line OD. Table 1 shows descriptive statistics for the radiographic measurements of the A1 group and A2 group, and Table 2 shows the descriptive statistics for the B1 group and B2 group.

**Table 1:** Descriptive statistics for radiographical mesurements of A1 and A2 group

Variables	**Group A1**		Group A2		Statistic(t)			*P* value	
	**(*n*** **= 180)**		**(*n*** **= 172)**						
MB (mm)	11.21 ± 1.62		10.85 ± 1.47		2.180			0.029	
NC (mm)	16.55 ± 1.67		15.96 ± 1.38		3.604			0.000	
BC (mm)	10.44 ± 1.33		9.95 ± 0.98		3.921			0.000	
OD (mm)	5.39 ± 0.78		5.22 ± 1.21		1.573			0.117	
PE (mm)	9.19 ± 1.52		9.02 ± 1.29		1.128			0.260	

**Table 2:** Descriptive statistics for radiographical mesurements of B1 and B2 group

**Variables**		**Group B1**		**Group B2**		**Statistics**	***P*** **value**		
		**(*n*** **= 165)**		**(*n*** **= 187)**					
MB (mm)		10.21 ± 1.42		11.65 ± 1.39		3.072	0.002		
NC (mm)		15.62 ± 1.29		16.08 ± 1.62		2.921	0.004		
BC (mm)		9.81 ± 0.85		10.19 ± 1.05		3.700	0.000		
OD (mm)		5.18 ± 0.71		5.31 ± 0.91		1.480	0.139		
PE (mm)		8.82 ± 1.19		9.21 ± 1.48		1.316	0.189		

The numbers of lines MB, NC, and BC in the A2 group were lower than those in the A1 group (*P* < 0.05; Table 1). There was no significant difference in terms of line OD or line PE between the A1 group and the A2 group (*P* > 0.05; Table 1).

There was no significant difference in the comparison of line OD or line PE (*P* > 0.05; Table 2) between the B1 group and the B2 group. The line MB, line NC and line BC in the B2 group demonstrated significant higher compared to those in the B1 group (*P* < 0.05, Table 2).

## Discussion

Tibial plateau fractures involving the posterolateral quadrant are special kinds of fractures that account for approximately 7-15% of tibial plateau fractures and are among the most difficult to treat [14–16]. In addition, as the fragment is in the posterior part of the lateral tibial plateau, the fracture is hidden on X-ray, and the rate of misdiagnosis is high during emergency treatment [17]. Anatomical repositioning and strong internal fixation are necessary to achieve functional rehabilitation in patients with intra-articular fractures. Misdiagnosis and inappropriate treatment can lead to serious joint deformity and functional impairment.

### Deficiencies of various approaches for accessing posterolateral tibial plateau fractures

Currently, several types of alternative surgical approaches have been described for plating posterolateral fractures of the tibial plateau, including the posterior approach, the posterolateral approach with fibular osteotomy, the modified posterolateral approach, and the traditional anterolateral approach [1, 4, 7, 18, 19]. Most orthopedic surgeons are familiar with the traditional anterolateral approach for its simple manipulation. Nevertheless, direct visualization and fixation of the posterolateral fragments are usually insufficient due to its covering by the caput fibulae and ligamentous tissues; thus, the traditional anterolateral approach is less commonly used for treating posterolateral fractures of the tibial plateau [20, 21]. The posterior approach, posterolateral approach with fibular osteotomy, and modified posterolateral approach have the merits of allowing full visualization of the posterolateral quadrant of the tibial plateau and effective manipulation of posterolateral fracture fragments, thereby achieving anatomical reduction and rigid fixation [5, 18, 21, 22]. However, all these approaches require intraoperative dissociation of bundles of blood vessels and nerves, which not only prolongs operation time but also increases the risk of iatrogenic neurovascular injuries [9, 23]. Moreover, nonunion or malunion at the osteotomy site is a risk associated with fibular osteotomy [4]. In addition, neither the posterior nor the posterolateral approaches could be performed to plate anterolateral tibial plateau fractures since many cases were combined with split wedge fragments of the lateral tibial plateau, including those of Schatzker types II, V, and VI. Other disadvantages were associated with extensive surgical trauma, prolonged learning curves, and difficult withdrawal of the internal fixator during the second operation.

### The preponderance of the supra-fibular-head approach

To seek out an effective, simple, and safe surgical approach, our team studied the supra-fibular-head approach, which has been proven to be anatomically feasible in human cadavers and therapeutically validated in clinical practice [10, 11, 21]. This approach was designed according to the anatomical features of a certain amount of space above the fibular head and the relaxation of the lateral collateral ligament with the knee flexed. First, the incision was made closely along the anterior border of the fibular collateral ligament. Then, the space between the fibular collateral ligament and lateral tibial plateau along the superior edge of the fibular head with knee flexion to approximately 60° was dissected. Next, the fibular collateral ligament and hamstring tendon could be mobilized to retract posteriorly using a retractor, and the posterolateral fragments of the tibial plateau could be fully observed with further maneuvers of mild internal rotation and tibial inversion.

Based on our clinical practice, we have identified several benefits of the trans-fibula-head approach, including the following: (1) a safe approach, no vital neurovascular structure or stable structures of knee joint are encountered; (2) needless dissection of neurovascular and muscular structures with shorter operation times, less trauma, and less bleeding volume; (3) exposure of the whole lateral articular cavity and meniscus via a submeniscal approach, which can be used for managing meniscus injuries simultaneously; (4) direct visualization of the anterior cruciate ligament and lateral collateral ligament, enabling the treatment of ligament injury, as well as the proximal tibial fractures; (5) full exposure of the lateral tibial platform, enabling the confirmation of articular surface reduction to reduce long-term severe complications, such as the post-traumatic osteoarthritis [24]; (6) the internal fixation can be safely removed with a lower risk of neurovascular injury after the fracture heals.

### Significance of morphometric data on the superior space of capitulum fibulae

For the treatment of a posterolateral fracture of the tibial plateau, through the trans-fibular-head approach, three basic conditions must be fulfilled: firstly, adequate access to the posterolateral fragments of the tibial plateau; secondly, simple, and effective reduction and internal fixation in the space above the capitulum fibulae; and thirdly, no interference with the lateral meniscus, medial meniscus, or fibular collateral ligament by the internal fixator. Hence, the space above the capitulum fibulae was the emphasis of this study; this space included the bone-cartilage region (the space between the articular surface of the capitulum fibulae and the cartilage of the tibial plateau) and the bone-ligament region (the space between the lateral margin of the lateral tibial plateau and the fibular collateral ligament). In our study, we found that the shortest distance in the bone-cartilage region was between the superior border of the articular surface of the fibular head and the articular surface of the lateral tibial plateau, that is the line MB. Similarly, in the bone-ligament region, the shortest distance was between the lateral tibial plateau and the medial margin of the fibular collateral ligament, that is the line OD. Therefore, the values of the line MB and OD were decisive factors in determining the thickness and width of the horizontal arm of the anatomical plate used in the space above the capitulum fibulae.

Since cartilage is not visible on CT scan, the line MB measured by CT image is the distance between the bones, which ranges from 7.7 to 12.8 mm, with an average of (10.96 ± 1.39) mm. The average thickness of lateral tibial plateau cartilage in adults is 2.7 mm, and the average thickness is greater in males (3.09 ± 0.13 mm) than in females (2.61 ± 0.09 mm) [25]. Therefore, the distance between the articular surface of the capitulum fibulae and the cartilage of the lateral tibial plateau ranges from 10.4 to 15.5 mm, which is similar to our previous anatomic data [10]. In addition, the value was positively associated with height (*P* = 0.001, *R* = 0.17), and in adults taller than 155 cm, the values were greater than 10 mm. The mean OD was (5.41 ± 0.97) mm (ranging from 3.2 to 6.3 mm), the minimum of which was significantly larger than the general thickness of the anatomical plate (2–3 mm). Therefore, there was ample space to place the plate in the bone-ligament region without affecting the stretching of ligaments in terms of the thickness of the plate.

Posterolateral condyle fracture of the tibial plateau is a special type of fracture that is not classified according to the Schatzker classification. Recently, based on 3-D CT images, the classification, diagnosis, and treatment of tibial plateau fractures have been further standardized and developed. Luo et al. divided the tibial plateau into three columns: the lateral, medial, and posterior columns [3]. Hua et al. further expanded on this concept by proposing the “four-column”, which subdivides the posterior column into posteromedial and posterolateral columns [26]. In this study, we divided the lateral tibial plateau into three regions, which further refined and supplemented the previously mentioned lateral and posterolateral columns. This is highly important for selecting a surgical approach, guiding intraoperative reduction and fixation techniques, and designing anatomical locking plates for the trans-fibular-head approach. For lateral tibial plateau fractures involving regions I and II, region III could be routinely exposed through the trans-fibular-head approach. Region II could be exposed by dissecting the space between the fibular collateral ligament and the lateral margin of the lateral tibial plateau, and the fibular collateral ligament could be mobilized to retract posteriorly, exposing region I, that is, the posterolateral condyle of the tibial plateau. In region I, which was involved in the fracture, with or without region II, the depressed fracture fragments in region I could be exposed by “window” osteotomy in region III or intraarticular osteotomy in region II, providing poking reduction, bone grafting, and internal fixation under direct vision.

**Fig. 4:**
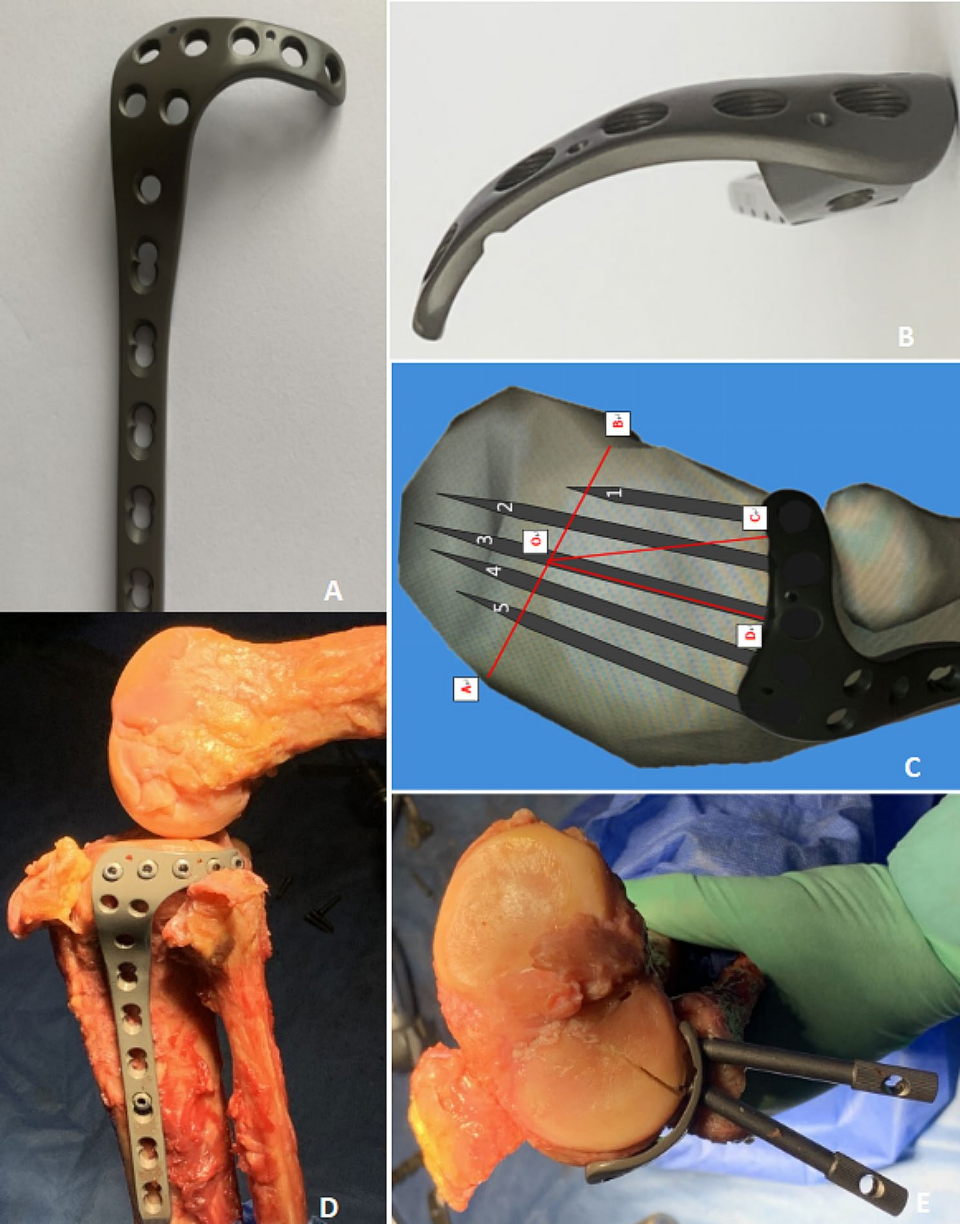
**A**, our designed anatomic plate is L-shaped with five holes on the horizontal arm. **B**, A view of the head of the anatomic plate. The head of the anatomic plate is a curved structure that can encompass the entire lateral tibial plateau. **C**, A schematic diagram showing the raft fixation above the fibular head. Mark **A** is the centre of the leading edge of the tibial plateau, mark B is the centre of the trailing edge of the tibial plateau, mark O is the centre of the tibial intercondylar eminence, mark **C** is the posterior margin of the articular surface of the fibular head, and mark **D** is the anterior edge of the articular surface of the fibula head. BOC represents Region I, DOC represents Region II, AOD represents Region III. Screw 1 and 2 can provide effective subchondral support for the posterolateral bone fragments (Region I), and the other three screws can properly fix the fragments in RegionIIand Region III simultaneously. **D**, **E**, Autopsy of a fresh specimen showing the simulated implantation of the anatomic plate and screw

After fracture reduction and bone graft support using the trans-fibular-head approach, the optimal internal fixation should encircle the lateral tibial plateau in an arc and simultaneously region I-III being fixed. The horizontal arm should provide effective support for fracture fragments in region I, and the safe width should be 9 mm (the bone-cartilage distance, MB + cartilage thickness). Region II is a trapezoidal structure with a length measuring more than 8 mm (the minimum value of line BC) and an anterior width of less than 16 mm (the bone-cartilage distance, NC + cartilage thickness). Moreover, screws in this region should be inserted into the posterolateral fracture fragments to strengthen the supporting effect. As the main body in region III, the plate should be anatomically designed and extended to the tibial shaft. Currently, the L-shaped Pilon anatomical plate is mostly used in our clinical practice, which is relatively suitable for managing fracture fragments in regions II and III. However, due to constraints of the width and length of the horizontal arm of the available market plate (11 mm in width, 38 mm in length, 2 mm in thickness), the plate cannot completely adhere to region I; thus, the direct support of the plate in region I is lacking. To address this issue, a proximal locking screw should be inserted in the posterolateral direction to strengthen the support of the posterolateral condyle to compensate for the limitations of biomechanics. However, biomechanical research found that one locking screw cannot offer appropriate support to the posterolateral fragment, potentially leading to fixation failure [27]. To address these issues, the authors designed and patented the anatomical plate for the trans-fibular-head approach (China Patent Number: ZL201220528187.9). This anatomical plate has a curved head, that can encompass the entire lateral tibial plateau. The head of the plate wraps around Region I, two screws can provide effective subchondral support for the posterolateral bone fragments, and the other three screws can fix the fragments in Region II and Region III simultaneously. Through the encircling support of the plate head and the raft support of the subchondral screws, the fixation of the posterolateral bone fragments can be strengthened. Figure 4 shows our designed anatomical plate.

Some limitations of the present study need to be acknowledged. The study population involved in this study was relatively young; perhaps, morphometric information about the area above the fibula capitulum may differ among older patients due to the influence of osteoarthrosis of the knee [28]. In addition, further testing is needed to verify the biomechanical performance of our designed plate for managing posterolateral tibial plateau fractures.

## Conclusion

The bone-cartilage region and bone-ligament region above the fibular head were the anatomical basis for designing the supra-fibular-head approach. The three-dimensional CT partition and the parameters of each region were crucial for providing guidance for designing the anatomical plate for the trans-fibular-head approach. Based on the above vital data, the space above the fibular capitellum was ample enough to place the plate and the posterolateral bone fragments could be strengthened by the encircling support of subchondral screws in the plate head. At present, long-term follow-up studies with a large cases of tibial plateau fractures involving posterolateral quadrant are now being undertaken to investigate the clinical effect of our anatomical plate via the trans-fibular-head approach. Perhaps, it will have great potential for widespread clinical application in treating posterolateral fractures of the tibial plateau, and provide a reference method for the treatment of posterolateral fractures of the tibial plateau.

## Data Availability

The datasets used and/or analysed during the current study are available from the corresponding author or co-author on reasonable request.
